# Beam-steering metasurfaces assisted coherent optical wireless multichannel communication system

**DOI:** 10.1515/nanoph-2023-0352

**Published:** 2023-07-17

**Authors:** Jin Tao, Quan You, Chao Yang, Zile Li, Liangui Deng, Mian Wu, Ming Luo, Lin Wu, Chao Li, Zichen Liu, Zhixue He, Xi Xiao, Guoxing Zheng, Shaohua Yu

**Affiliations:** State Key Laboratory of Optical Communication Technologies and Networks, China Information Communication Technologies Group Corporation (CICT), Wuhan 430074, China; National Information Optoelectronics Innovation Centre, Wuhan 430074, China; Peng Cheng Laboratory (PCL), Shenzhen 518055, China; Wuhan Institute of Quantum Technology, Wuhan 430206, China; Electronic Information School, Wuhan University, Wuhan 430072, China; School of Microelectronics, Wuhan University, Wuhan 430072, China

**Keywords:** metasurface, optical broadcasting, optical wireless communication

## Abstract

The metasurface based beam-steering devices with the advantages of large steering angles, arbitrary channels and ultra-compactness have played an important role for data allocation and exchange in the optical wireless communication. However, the current metasurface based optical wireless communication systems are mainly on intensity modulation and direct detection (IM/DD), which shows a relative lower transmission capacity, lower received optical signal-to-noise ratio (OSNR) and complexity of system. In this study, a bidirectional multichannel optical wireless system enabled by a polarization independent metasurface with coherent modulation and reception is designed and experimentally demonstrated, which exhibits exclusive 100 Gbps coherent optical signals to multiusers with their own wavelengths, 2 m free space distance and field of view of 20° × 20°. In addition, the proposed system can support optical broadcasting system with capacity of 900 Gbps. The demonstrated metasurface assisted optical wireless communication system merges the optical coherent communication techniques and emerging concept of metasurface, which reduces the complexity and cost of the system while contributing a high transmission capacity, opening a new avenue for high performance optical wireless communications.

## 1 Introduction

With the fast development of mobile internet scenario and the Internet of Things, the current wireless communication traffic is becoming more vulnerable due to the available radio spectrum is being rapidly exhausted. Optical wireless communication (OWC) has been considered an attractive solution or alternative for alleviating the congesting radio spectrum due to the huge spectrum resource and immunity against electromagnetic interference [1]. Especially in near infrared range, it is compatible with the existing well-established optical communication system with a huge bandwidth and does not depend on the switch state of the illumination source. Beam steering technology with good directivity and flexibility plays an important role for data transmission and allocation in the OWC. Conventional methods including microelectromechanical system (MEMS) [2–4], spatial light modulator (SLM) [5–7], and diffraction gratings [8–10] have been intensively investigated and applied in indoor OWC system with high-access number and high-capacity. Recently, the concept of the metasurface consisting with subwavelength ultrathin resonant nanostructures has been introduced into the field of optical communications [11–14] and shown strong wave control ability in OWC [15, 16]. A dielectric transparent metasurface was employed to convert two orthogonal linearly polarized Gaussian beam from fundamental mode to higher order fiber modes for spatial mode multiplexing with 100 Gbps data transmission at telecommunication wavelength, where spatial mode increases a degree of freedom for information modulation [12].

A metallic metasurface working as a polarization beam splitter was experimentally applied for a point to point 1D infrared wireless link with 20 Gbps over a 35° beam steering angle [15]. Very recently, we mass manufactured beam-steering metasurface for a full-duplex optical wireless-broadcasting communication system, which exhibits beam steering angles up to ±40°, 14 broadcasting channels with capacity for downstream and upstream links up to 100 and 10 Gbps for each user channel, three operating modes for flexible signal switching, and metadevice dimensions as small as 2 mm × 2 mm [16]. However, the modulation format of the high-speed signal in these schemes was intensity modulation format such as non-return-to-zero (NRZ) and 4-level pulse amplitude modulation (PAM-4). As the maximum launched optical power in free space is limited under 10 dBm for the eye safety limits according IEC 60825 and ANSIX136 standards, the low receiver optical power will limit transmission capacity. Therefore, optical amplifiers must be introduced to meet the requirements of receiving sensitivity, which will increase the complexity and the cost of the OWC system. In addition, in the wavelength division multiplexing (WDM) system, the optical signal-to-noise ratio (OSNR) of each wavelength decreases as the increasing number of wavelength due to the limited transmission power and optical filters are required for demodulating signals with different wavelength [16].

Coherent optical communication with coherent modulation and heterodyne detection has the technical advantages of longer transmission distance and larger transmission capacity, which has received much attention and has been applied both in the academic and industry field [17–21]. Compared with intensity modulation and direct detection (IM/DD), optical coherent modulation and reception techniques benefit the high capacity for OWC system, which can recover signals of different wavelengths without optical filter and has low receiving sensitivity. However, to the best of our knowledge, ultra-high capacity metasurface based OWC system with coherent signals has not yet been developed.

In this work, we proposed and experimentally demonstrated a bidirectional coherent optical wireless multichannel communication system assisted by a polarization insensitive beam-steering metasurface. The coherent reception-based 100 Gbps real-time dual polarization quadrature phase shift keying (DP-QPSK) signal free-space are distributed to 9 users over 2 m distance without EDFAs and optical filters at reception side. The polarization dependent loss (PDL) of the metasurface is less than 1.2 dB. The bit error rates (BERs) for all of the users are below the soft decision forward error correction (FEC) limit of 2.4 × 10^−2^. Each user can flexibly receive both all wavelength signals for the meta-OWC system and their own wavelength signals. The proposed metasurface assisted OWC system merges optical coherent optical communication and emerging metasurface, which reduce the complexity and cost of the OWC system while maintaining a high data-rate, suggesting a promising architecture for future high-performance wireless communications.

## 2 Beam-steering metasurfaces and coherent OWC system implementation

**Figure 1:**
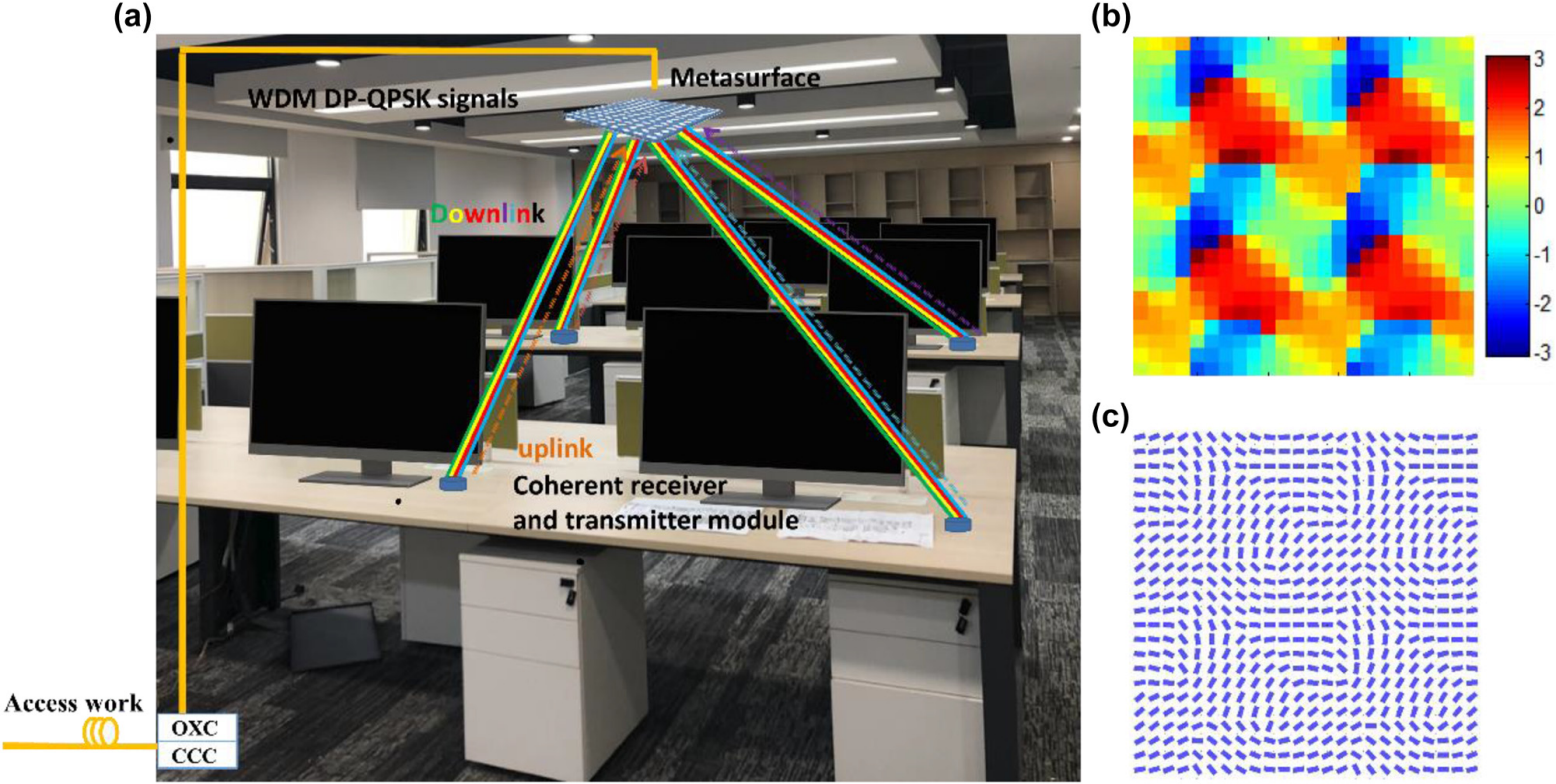
Conceptual illustration and design for beam-steering metasurfaces assisted coherent optical wireless multichannel communication system. (a) Schematic of beam steering metasurface assisted bidirectional optical wireless communication system by using coherent modulation and reception. (b) Partial phase distribution (24 × 24 pixels) of the metasurface. (c) Corresponding layout of the designed metasurface consisting of Si nanobrick arrays with different orientation angles.

Figure 1 illustrates the schematic diagram of beam steering metasurface assisted multichannel optical wireless communication system using coherent modulation and reception. In the house or building, the metasurface device was installed on the ceiling as an optical base station. The base station is arranged with the wavelength division multiplexing (WDM) DP-QPSK signals by the optical cross connector (OXC) which connected to a communication control center (CCC). The WDM light signals through the fiber were incident on the metasurface. Optical coherent signal coming from the optical fiber access network is then sent to the multi-users via the metasurface device. Due to the Pancharatnam–Berry phase modulation with centrosymmetric design, the output beam will project to a centrosymmetric sub-beam array with polarization independence. The divided beams are then transmitted through the indoor free space to the multi-users and received by the coherent receiver at user terminals. The users can choose to receive coherent signal from the metasurface with their own wavelength or all WDM signals as an optical wireless broadcasting system. As the designed metasurface is optical reciprocal for the beam transmission, the system supports data upstream link transmission into communication control center for each user as indicated in dash line in Figure 1, in which each user can use a wavelength as optical carrier for his own signal. Figure 1(b) shows the partial phase distribution of the metasurface. Figure 1(c) shows the corresponding layout of the designed metasurface with different orientation distribution of nanobrick.

**Figure 2:**
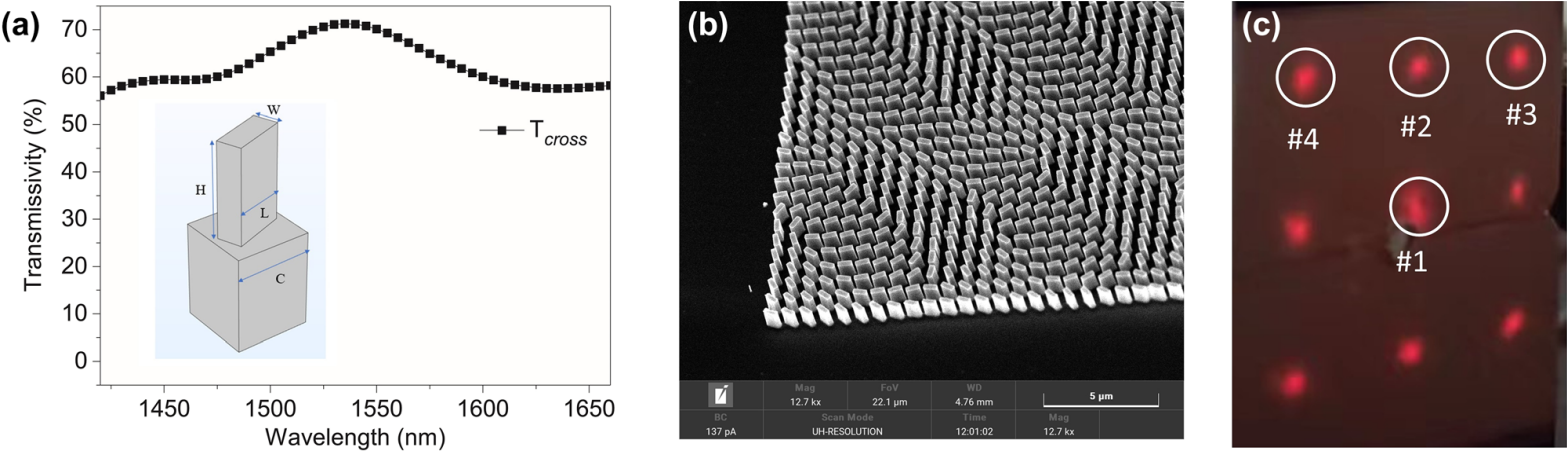
Design and characterization of beam-steering metasurface. (a) Simulated transmission spectrum of the cross-polarized (*T*_cross_) component for under normal circularly polarized light incidence. The inset: schematic diagram of the silicon-based nanostructure unit-cell. (b) Partial SEM image of the fabricated metasurface. (c) Beam spots distribution on the IR sensitive card after the metasurface.

In this work, we used c-Si material to design and fabricate the transmissive dielectric metasurface, which consists of nano-brick structures with same dimension (cell size *C*, length *L*, width *W* and height *H*) and different orientations as shown in the inset of Figure 2(a). The refractive index of the silicon wafer is 3.47. The polarization conversion efficiency (PCE) is used to characterize the optical efficiency of Pancharatnam–Berry phase (P–B phase) modulation. By choosing the geometric dimension of the nano-brick structure, the PCE can be optimized for the design of the transmissive dielectric metasurface. We used the CST STUDIO SUITE to optimize the performance of the nanostructure unit-cell by simulating the transmissivity of the nanostructure, and worked out an optimized nanostructure (*C* = 800 nm, *L* = 600 nm, *W* = 260 nm and *H* = 1000 nm) with a high PCE (*R*_cross_ = 70 %) at the working wavelength of 1550 nm [Figure 2(a)]. Then we employed the Gerschberg–Saxton (GS) algorithm to record a phase-only holographic 3 × 3 spot array into the silicon metasurface with the Dammann gratings scheme. As the opposite phase delays under left-handed circularly polarized (LCP) and right-handed circularly polarized (RCP) incidences, the holographic images generated under LCP and RCP incidences are centrosymmetric. So designed beam-steering metasurface is polarization insensitive, which is crucial for the coherent optical wireless communication as two orthogonal linear polarized beams are employed for coherent signal modulation. In addition, due to the P–B phase modulation, the metasurface works for a broadband response covering the well-established all *S*, *C*, and *L* bands of fiber communications.

The metasurface was finally designed to generate a sub-beam array with field of view of 20° × 20° in the Fourier space. The silicon based metasurface with the size of 801.6 × 801.6 μm^2^ was manufactured by the standard electron beam lithography followed a dry etch process and only single-step lithography process is required during the fabrication. Figure 2(b) shows the scanning electron microscopy (SEM) of the silicon based metasurface, which consists of periodic nanobricks with different orientations.

Then we characterized the optical beam steering performance for the polarization independent metasurfaces. The metasurface is normally illuminated by a collimated laser beam at the operation wavelength of 1550 nm. After the metasurface, the incident beam is projected into 3 × 3 beam spots with an extending angle of 20° × 20°, as shown the beam distribution on the IR sensitive card in Figure 2(c). As the cell periodic size of the metasurface is much smaller than that of the conventional optical elements, one can see from the figure the sub-beams are uniform with eliminating high diffraction orders and large beam-steering angles. The intensity of centric sub-beam is higher than the others because of the residual zero order in the hologram computation. The measured transmission efficiency is 17 %, which is lower than expected value. This is due to the influence of manufacturing errors, interface reflection and surface roughness of the silicon wafer. The efficiency of the metasurface can be further improved by increasing the size of the metasurface using standard complementary metal-oxide-semiconductor (CMOS) process and adding the antireflection layer on the metasurface.

**Figure 3:**
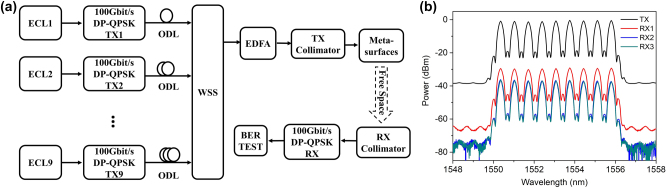
Experimental system configuration and spectra for beam-steering metasurfaces assisted coherent optical wireless multichannel communication system. (a) Experimental setup of Gbps optical coherent wireless multichannel communication enabled by beam-steering metasurfaces. (b) The launched and received spectra for 9 typical users for metasurface based optical coherent wireless communication system. Each wavelength is modulated with 100 Gbps DP-QPSK signals.

We applied aforementioned polarization-insensitive metasurface to a coherent optical wireless multichannel communication system for the proof-of-concept. Figure 3(a) shows the experimental system configuration setup of coherent optical wireless multichannel transmission system based on coherent modulation and reception. Nine external cavity lasers (ECL) operate at wavelength of 1555.8 nm, 1555.12 nm, 1554.44 nm, 1553.76 nm, 1553.08 nm, 1552.4 nm, 1551.72 nm, 1551.04 nm, and 1550.36 nm, respectively, as shown measured launched spectra in the Figure 3(b). Nine channel signals of 100 Gbps DP-QPSK signals are generated by DP-QPSK integrated modules. All the optical signals are then spectral shaped and combined by a C-band wavelength selective switch (WSS). Finally, nine 100 Gbps DP-QPSK transmission signals are generated with the wavelengths from 1550.36 nm to 1555.8 nm. The modulated optical signals are then amplified by an EDFA into 20 dBm. As the measured transmission efficiency of the dielectric metasurface is 17.61 %, so the insertion loss of the device is 7.5 dBm, meanwhile the input optical beam is split into 9 sub-beams while introduce another 9.5 dB losses. So, the optical power of the 9 sub-beams is about (20–7.5–9.5) = 3 dBm, which is below eye-safe operation power. Then the optical signal is launched into free space by a transmitter collimator with a beam waist of 0.2 mm, after 2 m free space distance transmission the sub-beams were detected by nine receiver collimators with beam waist of 0.42 mm. Finally, the optical signals are detected by the DP-QPSK coherent module for BER calculation. According to principle of coherent reception, all optical signals with different wavelengths can be received in the electrical domain. Due to the limitation of bandwidth of electronic devices and large frequency spacing between different wavelengths, the signals can be output within the bandwidth range of the selected optical signal. Therefore, no EDFAs and optical filters are employed at the receiver side, which indicate that the high capacity optical wireless communication system is simple and lower-cost implementation.

We investigated the received sensitivity performance of proposed OWC system for three typical user distributions as seen in Figure 2(c). User-1, user-2 and user-3 correspond to the sub-beam spots with Nos. 1 to 3 respectively. From the Figure 3(b), it is noted that the attenuation of all wavelength has a good degree of consistency. As the metasurface was design based on the Pancharatnam–berry phase modulation, the optical phase was determined by the rotational angle of nanobrick. Therefore, the metasurface can work for a broadband wavelength range. However, diffraction angle and wavelength are correlated. In our design, the diffraction angle is 10° at the wavelength of 1550 nm. When the wavelength increases to 1555.8 nm, the diffraction angle will increase to 10.037°. Such a small amount of dispersion does not affect the communication performance. When the wavelength range becomes larger, it is necessary to consider the impact of dispersion, namely, the design of dispersion-compensating metasurfaces becomes crucial.

**Figure 4:**
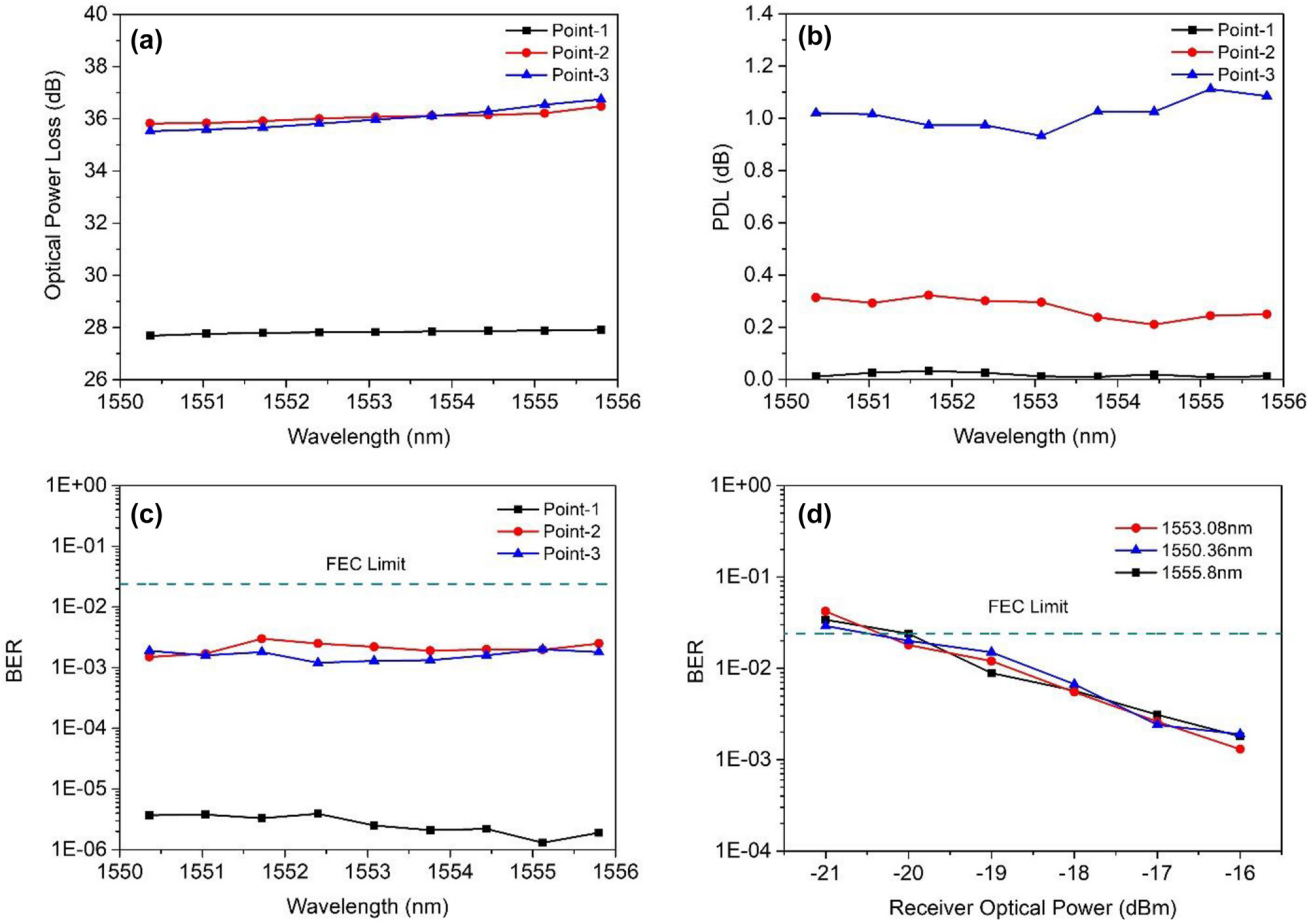
Experimental results of downlink transmission for beam-steering metasurfaces assisted coherent optical wireless multichannel communication system. (a) Measured losses of the nine wavelengths at point-1, point-2 and point-3, respectively. (b) Measured PDL of the nine wavelengths at point-1, point-2 and point-3, respectively. (c) BER performances of the 100 Gbps real-time DP-QPSK signal for all of the nine wavelengths at point-1, point-2 and point-3. (d) Performances as a function of ROP for point-3 with the wavelengths of 1555.8 nm, 1553.08 nm, and 1550.36 nm.

Figure 4(a) shows the measured losses of the nine wavelengths at point-1, point-2, and point-3, respectively. One can see that at point-2 and point-3, the values of the losses are in the interval of [35.5 36.7] dB. The values of the losses for the nine wavelengths at point-1 are around 27.9 dB, which are less than the value at point-2 and point-3 that is because the zero order diffraction will enhance the optical power at the point-1.

Then, we measured the PDL of three users located at point-1, point-2, and point-3, respectively as seen in Figure 4(b). The PDL at point-1, point-2, and point-3 are about 0 dB, 0.3 dB, and 1 dB, respectively. As the increasing of the distance between the chosen points to center of the metasurface device, the value of the PDL becomes larger, and all the PDL is contented to the coherent signal transmission.

Figure 4(c) shows the BER performances of the 100 Gbps real-time DP-QPSK signal for all of the nine wavelengths at point-1, point-2, and point-3. We can see that when the launch power is 20 dBm, the BER at point-2 and point-3 have the similar value at the level of 10^−4^. As the receiver power at point-1 is about 6 dB higher than that at point-2 and point-3, the BER value at point-1 are at the level of 10^−6^. Totally, all the wavelengths for the three users have the BER performance which below the FEC limit (BER level of 2.4 × 10^−2^). Figure 4(d) shows the BER performance as a function of received optical power (ROP) for point-3 with the wavelengths of 1555.8 nm, 1553.08 nm, and 1550.36 nm, respectively. One can see that the chosen three wavelengths have the similar performances, as the decreasing the ROP, the BER performance becomes worse. When the ROP is more than −20 dB, all wavelengths will get similar BER performance below the FEC limit. All the results shown in Figure 4(c) indicate that all of the nine users randomly located in the nine points can getting the 100 Gbps coherent signal from the metasurface with their own wavelength. In addition, the system can also support an optical wireless broadcasting system, which all users receive all wavelength signals of 900 Gbps.

**Figure 5:**
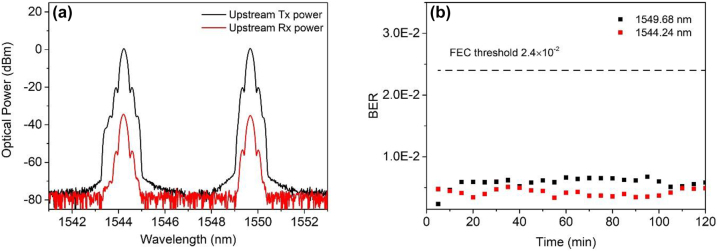
Experimental results of uplink transmission. (a) The launched and received spectra for two users in uplink transmission link at the operation wavelengths of 1544.24 nm and 1549.68 nm, respectively. (b) Stability test for points 2 and 3 in uplink transmission.

As the designed metasurface is optical reciprocal for the beam transmission, we chose 2 typical users (points 2 and 3) from the downstream user terminal and used the wavelengths of 1544.24 nm and 1549.68 nm as optical carrier for upstream link transmission. Figure 5(a) shows the launched and received spectra of upstream transmission for two users at the operation wavelengths of 1544.24 nm and 1549.68 nm, respectively. The optical power loss for the two users is ≈35 dB, which are similar to those of downlink transmission. We observe from the three BER curves that the metasystem has a similar performance as downlink.

Finally, we investigated the stability of the upstream data optical wireless communications system. A 2-h test is implemented, and the BER of 100 Gbps signal for points 2 and 3 were recorded every 5 min in a room temperature environment as shown in Figure 5(b). One can see that the BER was always below the FEC threshold. Therefore, the practical implementation for bidirectional coherent optical wireless multichannel communication system is feasible.

## 3 Discussion and conclusions

We have experimentally demonstrated a bidirectional WDM based coherent optical wireless communication system enabled by beam-steering metasurfaces, which utilizes the coherent WDM coherent and free spatial division multiplexing for high capacity and wide cover area. The transmission of real-time 100 Gbps DP-QPSK signal is successfully bidirectional transmitted over 2 m free space without EDFA at receiver for the nine users; the BERs for all of the users are below the soft decision FEC limit of 2.4 × 10^−2^, which reveals that the proposed system is feasible to the future indoor high-capacity networks. Compared with the preciously metasurface based OWC system [15, 16], the proposed coherent optical wireless system does not need EDFAs and filters at the coherent reception, which reduces the system complexity and cost. The system can support optical broadcasting system with capacity of 900 Gbps, where the user receives all WDM signal. In addition, within the eye safety power limit of 10 mW, the coherent modulation and reception technology guarantee a high received OSNR with a high capacity and longer transmission distance, which will also compensate the sacrificial efficiency of the metasurface.

In practical applications of OWC, tunable or active metasurfaces are necessary. Although there are many challenges in realizing tunable functions, actively-tunable metasurfaces based on various tunable materials and designs including phase change materials [22–24], transparent conducting oxide [25, 26], two-dimensional materials [27–29], metal hydrogenation [30, 31] and polarization state switching [11, 14, 32, 33] have been developed. Besides, an actively beam-steering meta-device can be realized by utilizing an ultracompact metasurface assisted with a spatial light modulator, which can significantly increase the beam-steering angle with user-defined dynamic beam-steering ability for intelligent optical wireless-broadcasting communication.

Overall, the proposed metasurface assisted coherent optical wireless multichannel communication system merges optical coherent optical communication and emerging metasurface technology, which utilizes the strong beam-steering ability of metasurface and advantage of coherent optical communications, which make metasurface beam-steering OWC more practical, suggesting a promising architecture for future high-performance wireless communications.
